# Clinical implication of hyperuricemia on the recurrence of atrial fibrillation after catheter ablation

**DOI:** 10.1002/joa3.13047

**Published:** 2024-05-11

**Authors:** Naoya Kataoka, Teruhiko Imamura

**Affiliations:** ^1^ Second Department of Internal Medicine University of Toyama Toyama Japan

To editor:

Hyperuricemia has been linked to a heightened incidence of atrial fibrillation (AF). Oseto and colleagues have demonstrated a significant elevation in serum uric acid levels among patients with persistent AF compared to those with paroxysmal AF.^1^ Moreover, the presence of post‐ablation hyperuricemia has been associated with the recurrence of AF in patients with persistent AF. However, several concerns have been raised.

Uric acid is primarily synthesized by xanthine oxidases, predominantly found in the liver, thus indicating a substantial influence of liver function on serum uric acid levels.^2^ In the authors' study, both uric acid and γ‐glutamyl transpeptidase levels were elevated in patients with persistent AF.^1^ Elevated γ‐glutamyl transpeptidase levels may be linked to alcohol consumption, which has been associated with the onset and recurrence of AF.^3^ It is highly recommended to adjust for these potential confounders to accurately assess the impact of serum uric acid levels on AF recurrence.

While the authors focused on the association between uric acid and left atrial remodeling,^1^ noteworthy that only a small fraction of xanthine oxidase is located in the left atrium.^2^ Did the author correlate serum uric acid levels with left atrial size, which may be more appropriate? Additionally, investigating the prognostic impact of xanthine oxidase, rather than uric acid, could yield more relevant insights.^4^

The clinical implications of predicting recurrent AF using post‐ablation data remain uncertain. Risk stratification of ablation candidates can be enhanced, allowing for more intensive ablation procedures, with reference to pre‐procedural risk factors instead of post‐procedural ones. Given their findings that serum uric acid levels were higher in patients with persistent AF compared to those with paroxysmal AF,^1^ post‐ablation elevated uric acid levels may simply reflect ongoing AF recurrence.

## CONFLICT OF INTEREST STATEMENT

Authors declare no conflict of interests for this article.

## Data Availability

This manuscript does not contain any data to be shared.
